# DNA Damaging Effects, Oxidative Stress Responses and Cholinesterase Activity in Blood and Brain of Wistar Rats Exposed to Δ^9^-Tetrahydrocannabinol

**DOI:** 10.3390/molecules24081560

**Published:** 2019-04-19

**Authors:** Nevenka Kopjar, Nino Fuchs, Suzana Žunec, Anja Mikolić, Vedran Micek, Goran Kozina, Ana Lucić Vrdoljak, Irena Brčić Karačonji

**Affiliations:** 1 Institute for Medical Research and Occupational Health, Zagreb HR-10001, Croatia; nkopjar@imi.hr (N.K.); suzana@imi.hr (S.Ž.); vmicek@imi.hr (V.M.); alucic@imi.hr (A.L.V.); ibrcic@imi.hr (I.B.K.); 2 University Hospital Centre Zagreb, Zagreb HR-10000 Croatia; Ninofuchs84@yahoo.com; 3 University Centre Varaždin, University North, Varaždin HR-42000, Croatia; goran.kozina@unin.hr

**Keywords:** acetylcholinesterase, antioxidative enzymes, brain cells, butyrylcholinesterase, genotoxicity, glutathione, comet assay, lipid peroxidation, white blood cells

## Abstract

Currently we are faced with an ever-growing use of Δ^9^-tetrahydrocannabinol (THC) preparations, often used as supportive therapies for various malignancies and neurological disorders. As some of illegally distributed forms of such preparations, like cannabis oils and butane hash oil, might contain over 80% of THC, their consumers can become intoxicated or experience various detrimental effects. This fact motivated us for the assessments of THC toxicity in vivo on a Wistar rat model, at a daily oral dose of 7 mg/kg which is comparable to those found in illicit preparations. The main objective of the present study was to establish the magnitude and dynamics of DNA breakage associated with THC exposure in white blood and brain cells of treated rats using the alkaline comet assay. The extent of oxidative stress after acute 24 h exposure to THC was also determined as well as changes in activities of plasma and brain cholinesterases (ChE) in THC-treated and control rats. The DNA of brain cells was more prone to breakage after THC treatment compared to DNA in white blood cells. Even though DNA damage quantified by the alkaline comet assay is subject to repair, its elevated level detected in the brain cells of THC-treated rats was reason for concern. Since neurons do not proliferate, increased levels of DNA damage present threats to these cells in terms of both viability and genome stability, while inefficient DNA repair might lead to their progressive loss. The present study contributes to existing knowledge with evidence that acute exposure to a high THC dose led to low-level DNA damage in white blood cells and brain cells of rats and induced oxidative stress in brain, but did not disturb ChE activities.

## 1. Introduction

The worldwide use of various forms of cannabis preparations has been known for centuries. Although many of them are taken for recreational purposes, today we are also witnessing an increased use of approved cannabis preparations for symptoms management in cancer therapies, neurological disorders and various conditions associated with chronic pain. The antiemetic, anticachectic, analgesic or antispastic effectiveness of some preparations which contain purified or synthetic cannabinoids Δ^9^-tetrahydrocannabinol (THC) and/or cannabidiol has also been well recognised by the world’s most important regulatory agencies as the European Medicines Agency, United States Food and Drug Administration and Health Canada [1]. Despite the accessibility of the approved medications, many patients still rely on the use of various forms of illegal crude cannabis preparations to relieve their symptoms. A considerable lack of information regarding safety, overall effectiveness, possible adverse effects and risks for the consumers of such preparations, regardless of being approved or illegal, speaks in favour of extensive research in that regard. 

The chief bioactive component of the plant *Cannabis sativa* L. is Δ^9^-tetrahydrocannabinol (THC) [2,3]. Its absorption and metabolism greatly depend on the route of delivery. The oral LD_50_ of THC in rats is reported to be 800–1900 mg/kg, depending on formulation, strain and sex [4,5]. Much of the orally administered THC undergoes first pass metabolism in the liver, resulting in the formation of 11-hydroxy-Δ^9^-THC (THC-OH). This psychoactive THC metabolite undergoes further oxidation to the inactive 11-nor-9-carboxy-Δ^9^-THC (THC-COOH) [6]. Both the parent THC and THC-OH readily cross the blood-brain barrier. As a lipophilic compound, THC quickly enters highly vascularised tissues like the liver, and tends to accumulate in body fat. THC metabolites are subjected to enterohepatic recirculation. They are excreted within days and weeks, largely in faeces [3,7].

The majority of THC effects are mediated through cannabinoid receptors: CB1 that predominate on neurons in the brain, spinal cord and peripheral nervous system, and CB2 that occur primarily in leukocytes and the immune system. Major CB1 functions include inhibition of neurotransmitter release in the central nervous system, while CB2 receptors are primarily responsible for modulation of cytokine release in the immune system [7]. 

Up to now, genotoxicity of THC as a single chemical has not been extensively studied, and the results are inconclusive. In the available literature, no other study focused on the same experimental model and the same array of assays that would offer a reliable answer regarding the potential DNA damaging effects of THC. The majority of existing reports on a rat model referred rather to the toxicity of *C. sativa* preparations [8,9,10,11,12] than to that of pure THC. However, the explanation of underlying effects becomes difficult since *C. sativa* contains plenty of other bioactive substances as well [13]. Contrary to approved pharmaceutical products, which contain known and defined THC contents [14], illicit preparations (especially so-called cannabis oils and butane hash oil) usually contain a very high THC content, sometimes over 80% [14,15,16]. That is why their consumers might experience various detrimental effects or become intoxicated, especially due to contaminants associated with *Cannabis* cultivation and processing (pesticides, mycotoxins, heavy metals) or toxic solvents used for production of highly concentrated preparations [17,18,19]. 

Taking into account a general lack of information regarding the detrimental effects of THC, we decided to carry out a study on a rat model focused on assessments of DNA damage in white blood and brain cells, as well as on oxidative stress-related effects of THC, and changes in cholinesterase activity in plasma and brain caused by acute and repeated THC exposure. This study represents a continuation of our research that focused on the evaluation of toxic effects of THC in vivo. The findings reported in our previous paper [20] suggested that administration of THC resulted in DNA damage in hepatocytes and provoked changes in levels of some functional liver markers and oxidative stress markers in exposed male Wistar rats. To further characterise the toxicity profile of THC and clarify its DNA damaging potential, in this study we estimated genome sensitivity of two other cell types, functionally and metabolically different from liver cells, following acute and repeated THC exposure.

To establish potentially harmful THC effects, we deliberately selected a high THC dose, comparable to doses found in illicit preparations. We cannot exclude possible (geno)toxic outcomes associated with the use of prescribed medical cannabis preparations, since this issue is still not fully explained and documented in the available literature. However, as such medications have a well-defined THC potency (usually not exceeding 20% THC), controlled quality, and are administered under supervision of medical professionals, it is expected that the possible adverse effects related to the administration of such preparations could be better managed and/or prevented. We hypothesized that acute and repeated exposure to a high THC dose could possibly be associated with impairment of the biochemical and DNA markers, which ultimately might reduce the overall fitness of the exposed rats. Since the majority of approved THC-based medications like dronabinol (Marinol, Syndros) and nabilone (Cesamet) are delivered orally [14,21,22,23], this study selected the oral route of THC administration. As THC in illicit preparations has usually been taken orally in lipid-based formulations [24], which leads to its higher bioavailability, to deliver THC to experimental animals we applied its oil formulation by oral route.

The extent of treatment-related DNA damage was studied using the alkaline comet assay, whose usefulness has been confirmed previously in studies on rat models [25,26,27,28,29,30,31]. This assay directly measures single-, and double-strand breaks in DNA, alkali-labile sites, and single-strand breaks linked to incomplete excision repair. It also allows for the detection of DNA-DNA or DNA-protein crosslinks [32,33,34,35,36]. To estimate lipid peroxidation, we used the thiobarbituric reactive substances (TBARS) assay, which relies on the ability of secondary products of lipid peroxidation and other reactive aldehydes to react with thiobarbituric acid (TBA) [37,38]. To establish the efficacy of defence against reactive oxygen species (ROS), we measured the level of glutathione that scavenges free radicals or binds to electrophilic sites on endogenous toxins and detoxifies them [39,40], and studied activities of two fundamental antioxidant enzymes superoxide dismutase (SOD) and catalase. Finally, the total antioxidant capacity in both of the tested matrices was evaluated using a ferric reducing antioxidant power (FRAP) assay [41]. In this study we also measured cholinesterase activities (ChE) as a valuable biomarker of possible impairments of the cholinergic system.

We expect that such an experimental approach could contribute new information valuable for risk assessments of THC, especially in the cases associated with the use of its highly concentrated preparations. The issues addressed in this paper could also be of potential use to medical practitioners whose patients use such preparations for recreational purposes or as supportive therapy to alleviate symptoms such as chemotherapy-induced nausea and/or vomiting [22], diarrhoea, abdominal pain and weight loss in inflammatory bowel disease [42] or different neurological conditions [43]. In spite of growing knowledge associated with the use of THC-rich preparations, this field of research is still controversial and requires an accumulation of a relevant amount of novel data.

## 2. Results

Exposure to THC at a daily dose of 7 mg/kg resulted in no deaths of treated rats or signs of systemic toxicity at any time-point of concern. The exposed rats experienced only a slight and non-significant time-dependent reduction in body weight gain, compared to controls (data not shown). No significant treatment-related changes in brain weights were observed. Considering that the animals selected for the experiment were in good physical shape, such results imply that their general homeostatic mechanisms efficiently compensated treatment-related distress to maintain overall fitness during the seven days of experiment.

### 2.1. DNA Damage in White Blood and Brain Cells

**Figure 1:**
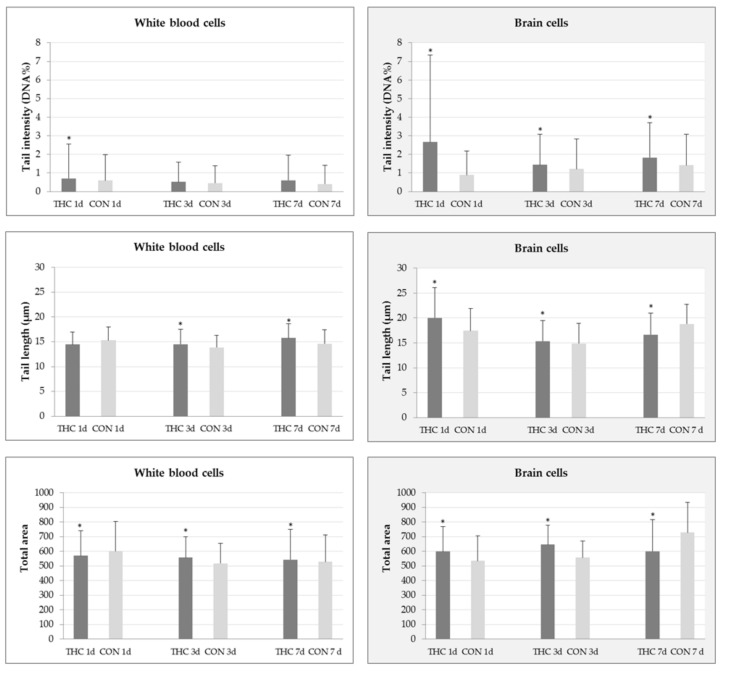
The levels of DNA damage measured using the alkaline comet assay in white blood cells and brain cells of male Wistar rats after 1-, 3-, and 7-day (d) treatments with Δ^9^-tetrahydrocannabinol (THC) and in the respective controls. The daily THC dose was 7 mg/kg b.w.; it was dissolved in sesame oil and administered per os. Control rats (CON) received the same daily volume of sesame oil as the THC group. Data represent mean ± standard deviation. There were five rats per group, and 200 comets per rat were scored on duplicate slides (altogether one thousand independent comet measurements per each experimental group were made). * *p* < 0.05 vs. control (Mann–Whitney U test).

Figure 1 reports results regarding the comet assay parameters determined in white blood cells and brain cells of rats exposed to THC at a daily dose of 7 mg/kg and corresponding controls. Background levels of primary DNA damage in either tissue were low. Following THC treatment, a low level of DNA damage was observed. The mean tail intensities measured in white blood cells at all time-points of interest were higher than in control rats, but this was statistically significant only for 1-day treatment. After 3- and 7-day treatments, THC-treated rats showed significantly increased mean values of tail length and total comet area compared to corresponding controls (Figure 1). The DNA of brain cells was more prone to breakage after THC treatment compared to DNA in white blood cells. A time-dependent increase of the mean tail intensity was observed. Additionally, the extent of DNA damage in brain cells of rats treated with THC for one and three consecutive days, perceived in terms of tail length and total comet area parameters, was significantly increased compared to respective controls (Figure 1).

### 2.2. Biochemical Markers of Oxidative Stress

Results on the levels of oxidative stress biomarkers measured in plasma and brain tissue are reported in Table 1. Acute oral administration of 7 mg/kg THC did not provoke substantial changes in the levels of oxidative stress biomarkers in rat plasma. In the brain tissue, a significant elevation of TBARS and glutathione (GSH) concentration, and drop in SOD activity was noticed. 

### 2.3. Cholinesterase Activities

The application of THC did not affect cholinesterase activity, either in the plasma or in brain tissue (Table 2). In the examined plasma samples, acetylcholinesterase activity (AChE) activity represents 65% of the total measured activity, and the remaining 35% was allocated to butyrylcholinesterase activity (BChE). Such a proportion was possibly due to the existence of free AChE in the plasma as well as partial haemolysis of rat erythrocytes prior to centrifugation of the whole blood. In the rat brain, AChE accounted for 90% of the total measured activity.

## 3. Discussion

The growing use of THC-rich preparations in various forms, both recreationally or prescribed by medicinal professionals, raises issues regarding their safety and risks for users, especially in vulnerable subpopulations as cancer or chronically ill patients. Since THC is often used with other conventional drugs and/or therapies, any information regarding its toxicity profile and potential to interact or enhance toxicity of other compounds is important and potentially valuable. The genotoxic potential of THC in vivo has been poorly investigated so far. Knowing the genotoxic potential of a compound is an important prerequisite to predict other potentially detrimental effects, both at the level of cells and an organism. In a recently published review, Reece and Hulse [44] stated that low doses of THC (<5 µg/mL or <5 µmol/L, or <1 joint/day) are usually not associated with genotoxic adverse outcomes. However, the doses of THC used by many of the patients who experiment with self-medication are usually considerably higher. It is hard to predict the genetic risks associated with such exposure, since the available literature offers limited evidence regarding THC effects at DNA level. In their review, Li and Lin [45] discussed the potential genotoxicity of commonly abused substances, including cannabis, and singled out its detrimental effects at DNA level. The recent literature offers only one in vitro study [46], which used the comet assay to estimate the genotoxicity of marijuana smoke condensates in lung cancer cells. Without doubt, the experimental approach applied in the present study is a novel one, as there is a general lack of accurate data in this regard. With a previous pilot study [20], our group was the first to apply the alkaline comet assay to evaluate THC genotoxicity on a rat model. In our previous paper, we showed that continuous 7-day oral THC exposure produced low-level DNA damage in hepatocytes. This paper provides evidence that brain and white blood cells responded with significant increases of DNA damage much earlier than hepatocytes, at the same tested THC dose, which points to their greater genome susceptibility. What further distinguishes the present study from the preceding one is the fact that in the first study we investigated the DNA damaging effects of anticancer drug irinotecan (IRI) and THC when administered to rats as single compounds and in combination. Particular emphasis was also given to mutual interactions of IRI and THC and their harmful effects in the rat liver. Bearing in mind a general lack of the information regarding THC genotoxicity, after extensive evaluation of data obtained using the comet assay and biochemical assays in rats exposed to single THC, we prepared the present paper as a separate publication, which we believe brings sufficiently relevant and new evidence regarding the outcomes of acute and repeated THC exposure on other rat tissues at a dose found in illicit preparations. This study provides original and valuable information regarding the levels of DNA damage in blood and brain cells of THC-treated rats along with novel information regarding THC impact on oxidative stress markers and cholinesterase activity that have not yet been familiar or reported before in relevant literature sources. 

The main aim of the present study was to investigate the genotoxic potency as well as the magnitude and dynamics of DNA breakage associated with THC treatment in vivo. The obtained results show the ability of both acute and repeated THC exposure to provoke measurable DNA damage in brain cells and white blood cells of rats. Increased DNA damage in brain cells of THC-exposed rats represents the most important finding of the present study and calls for concern, considering that it could impair viability of neurons, which ultimately might lead to their progressive loss and possible neurotoxic outcomes.

The observed differences in DNA susceptibility of white blood and brain cells might originate from the intrinsic differences between two tissues, and the efficacy of their inherent mechanisms which counteract damage. Since each of the tested tissues represents a specific matrix, the significance of obtained results will be discussed separately. 

In this study, slides for the alkaline comet assay were prepared using the samples of whole blood. The use of whole blood in the DNA damage analysis using comet assay was confirmed previously in a study by Al-Salmani et al. [47]. Considering that the comet method relies on recording of DNA damage in single nucleated cells [32,35], measured levels of genotoxicity represent the sum of DNA lesions inflicted in all white blood cells. As known, leukocytes are a heterogeneous population of highly differentiated nucleated cells that is comprised mainly of neutrophils (up to 80%), followed by lymphocytes, monocytes and eosinophils. When discussing the results, we have to bear in mind potential differences known to exist between these cell types. For instance, DNA repair capacities of white blood cells vary according to cell lineage [48], which might influence their genome susceptibility and sensitivity to genotoxic agents as well. Interpretation of the results about possible time-dependent genotoxicity should also focus on life-spans of white blood cells. As known, life-spans of neutrophils and monocytes are measured in hours/days, compared to lymphocytes which might circulate through the body for up to several months [49,50,51]. 

Even though the obtained results relied on measurements of three comet parameters, simultaneously calculated using the image analysis software, in this discussion we will place the main emphasis on tail intensity, as it is today considered the most useful parameter to describe the degree of DNA damage estimated by the comet assay. It points to the amount of DNA migrated in the tail and correlates with DNA break frequency. However, results obtained for two other comet parameters also contribute to the description of the overall THC genotoxicity, which could be characterised as being of low magnitude. As known, at low levels of damage comet tail length is also deemed a useful parameter [32]. Since it depends on the DNA loop length, after the comet tail is established, its length rapidly reaches a maximum and tends not to change [32,33]. The third comet parameter, i.e., total area, represents the overall surface area of the comet. Although not used as frequently as other comet parameters, its values can provide additional information on the extent of DNA damage that could further strengthen the significance of the comet assay findings. 

In the present study, we evaluated THC genotoxicity at three different time-points and found the highest level of DNA damage, in terms of tail intensity, after the 1-day exposure (Figure 1). Since such a short exposure period covers the life-span of all leukocytes, the mean tail intensity recorded after 24 h refers to the total DNA damage measured in all nucleated cells. However, explanation of DNA damage levels measured in blood samples taken after 3-, and 7-day exposure is somewhat more complex. Within this period, the lymphocyte pool cannot entirely renew, in contrast to rapid neutrophil/monocyte turnovers which happen daily. Therefore, we could say that the overall levels of DNA damage measured after repeated 3-, and 7-day THC exposure were largely affected by the “cumulative” responses of the lymphocyte genome to the applied treatment. In contrast, levels of DNA damage measured in neutrophils and monocytes possibly persisted, irrespective of the treatment duration, considering their constant replacement by fresh cells, and daily administration of the same THC dose. This assumption, indeed, has to be proven in future studies on isolated cell subpopulations.

A slight decrease in the mean tail intensity measured in white blood cells after 3-day THC exposure (Figure 1) might be a result of DNA repair in lymphocytes. However, judged from the perspective of an increased mean tail intensity, and the significantly increased mean tail length recorded after 7-day THC exposure, it is possible that DNA repair processes in lymphocytes finally became saturated. This could be, at least in part, explained by the quiescence state of lymphocytes, which is characterised by lower metabolic and many other activities [52]. When speaking about the genome sensitivity of other types of white blood cells, monocytes are very vulnerable, due to a lack of key proteins that take part in base excision and DNA double strand break repair [50,53]. Neutrophils, due to their very short life-span mostly do not respond to DNA damage via repair, and are intrinsically predetermined to die by constitutive apoptosis [49,54,55]. Taken together, from the DNA damage pattern observed in white blood cells during this experiment it could be concluded that THC produced a low level of DNA damage in white blood cells of rats, in spite of the relatively high daily dose administered.

Results of the alkaline comet assay show that 1-day exposure of rats to THC at 7 mg/kg resulted in detectable primary DNA damage brain cells (Figure 1). The highest maximum values of both comet parameters (32.51% DNA in tail and tail length of 54.58 µm vs. control values of 6.15% DNA in tail and tail length of 31.67 µm) recorded at this time-point suggest that some brain cells acquired large amounts of DNA lesions. Such a finding also points to the possible occurrence of apoptotic cells as a response to treatment. This is highly likely since THC-related neurotoxicity and apoptosis were previously described in several studies on brain cell cultures [56,57,58]. Worth mentioning, in these studies neurons were shown to be more susceptible to THC than glial cells. After repeated exposure, neurons also tended to accumulate THC and its metabolites, which led to adverse effects [58]. Given that the comet assay’s scoring criteria do not recommend a capture of nucleoids outside the limits of the image analysis software [59], it is possible that the real DNA damage level in the brain cells of rats at this time-point was even higher than we measured. For us it is reasonable to assume that a certain proportion of highly damaged cells “escaped” measurement, either as a consequence of measurement with an automated image analysis system or due to the critical steps of the alkaline comet assay (lysis, denaturation, electrophoresis), which contribute to the “wash out” of their fragmented DNA from the agarose microgel. That is why apoptotic cells often are lost during automated scoring if they are not recorded and scored visually as an addition to image analysis. 

Our results show that repeated THC exposure resulted in a time-dependent increase of tail intensity in the brain cells (Figure 1). In view of this, it is reasonable to conclude that THC at the tested dose and exposure conditions was genotoxic for rat brain cells in total, since the comet assay slides were prepared from dissected brain tissue that contained neurons, astrocytes and other types of glial cells. The existing literature suggests that neurons and glial cells respond differentially to genotoxic agents and oxidative stress [60,61] and possess different capacities for DNA repair [30,48]. Although neurons retain the same DNA repair systems as other eukaryotic cells (nucleotide excision, base excision, mismatch and double strand breaks repair), the repair of lesions in their DNA is much slower than in dividing cells [62]. Accumulation of unrepaired lesions might impair transcription and protein synthesis, which finally may trigger cell death. 

To obtain more specific explanations regarding the susceptibility of a particular cell type, the comet assay procedure might be coupled with cell separation and characterisation. However, as such procedures could represent a source of additional DNA damage detectable by the comet assay, to avoid false positive results, such an approach was not used in the present experiment.

Since the present study was a pioneer in the assessment of potential THC genotoxicity on rodent white blood and brain cells using the comet assay, it is not possible to draw a parallel between our findings and other literature sources. We can only mention the conclusions of our previous comet assay study where the same THC dose was used [20]. They suggest that continuous 7-day oral exposure of rats to THC caused low-level DNA damage in liver cells.

Considering that the alkaline comet assay identifies a wide array of lesions, we cannot define the quality and types of DNA lesions produced by THC treatment, or mechanisms behind the detected genome damage in white blood and brain cells of treated rats. However, without a doubt, the extent of DNA damage observed in the THC-treated rats was influenced by the exposure design. During the study, rats repeatedly received THC for seven days. This treatment approach led to a continuous delivery of new quantities of the tested chemical, which is extensively metabolised into many metabolites with a different half-life, and subjected to slow turnover and clearance from the organism [2,3,6,7,63]. Due to high lipophilicity, a part of the delivered THC also tends to accumulate in body fat, and slowly releases leading to “reintoxication” [64], producing potentially DNA-reactive compounds. We propose that the level of DNA damage measured after treatment is a sum of the lesions caused by mutual direct and indirect effects of parent THC, all of its metabolites, different reactive free radicals and secondary ROS. In addition, a part of induced DNA damage also depends on DNA repair processes, which might produce a certain quantity of further damage. Besides, after seven days a kind of balance between the formation and repair of DNA damage can be reached, which also influence the values detected by the alkaline comet assay. 

Since our study was limited by the choice of a single THC dose, the possible harmful effects of THC at DNA level should be further investigated on a wider range of doses. Taking into account all of the facts acknowledged in the available literature sources and our own observations, we believe the issue of THC genotoxicity—due to controversial and inconsistent results—has to be further studied. Information provided in the present study could also be potentially interesting to other researchers, especially those experienced in the application of a comet assay.

The outcomes of the comet assay motivated us to further investigate the potency of the tested THC dose to provoke oxidative stress in blood and brain cells. According to the literature, this is likely high, as some studies [65,66,67,68] associated THC exposure with impaired mitochondrial function, which results in enhanced production of hydrogen peroxide. Previous studies about THC (or *C. sativa*) effects on oxidative stress markers in rodents relied on various free radical-related parameters and revealed inconsistent results [8,20,65,69,70,71,72].

Considering the detected levels of DNA damage after 1-day THC exposure, we decided to assess the changes of oxidative stress biomarkers only at this time-point. Although this could be a potential limitation of the present study, a similar experimental design on a rat model was previously successfully applied by Costa and Colleoni [65], who tested an even higher THC dose (10 mg/kg), but using another array of assays. 

We found that acute exposure of rats to 7 mg/kg of THC resulted with a detectable increase in the concentration of TBARS in the brain. In spite of the potential lack of specificity of the applied TBARS assay, this result is an indication of possible lipid peroxidation.

Considering the essential role of glutathione in the cellular defence, our study also measured its levels after THC treatment. The obtained results show that acute THC exposure elevated brain GSH level (Table 1). Based on the existing knowledge on glutathione metabolism and cycling, the observed rise in brain GSH level in THC-treated rats, compared to their controls, could be associated with either treatment-related GSH production in brain or the uptake of GSH from plasma. The former is quite possible, since plasma GSH levels in THC-treated rats slightly lowered compared to controls (Table 1). As known, plasma GSH plays an important role in brain GSH homeostasis [73]. 

After single oral exposure to THC we found a slightly increased catalase activity and decreased SOD activity in brain. According to the literature, both SOD and catalase activities in rats tend to increase with longer exposure, and also after THC delivery via intraperitoneal route [72].

FRAP values, which remained within the control levels both in plasma and brain after a single administration of THC (Table 1) indicate that compensatory homeostasis among the different cellular antioxidants efficiently counteracted potential imbalances caused by acute treatment with the tested compound.

The results regarding oxidative stress markers could be, at least in part, explained by the oral route of THC delivery which was used in the present study. It is well known that oral administration of THC leads to its slow absorption. This type of delivery also allows for a greater removal of THC by the liver and more abundant formation of the active metabolite, THC-OH, which easily crosses the blood-brain barrier. As the brain extracts a large fraction of the drug present in the circulation, brain levels of the metabolite tend to gradually rise, while at the same time its plasma levels decline [7]. This largely explains the discrepancies between plasma and brain TBARS levels we found following oral THC delivery. In a similar study, Costa and Colleoni [65] found that a single intraperitoneal administration of THC at 10 mg/kg enhanced the energetic brain metabolism in rats, which resulted with increased lipid peroxidation. They associated the observed phenomena with increased mitochondrial oxygen uptake via the CB1 cannabinoid receptor.

The cellular damage by oxidative stress could have a resultant effect on loss of cognition and altered neurotransmitter system [74]. It is also known that acute THC effects are related to disturbed functioning in performance and cognitive tasks (reaction time, motor coordination and attention, learning perception) [75,76]. Since the central cholinergic neurotransmission in the brain is crucial for cognitive functions including learning and memory formation [77], we used ChE as a potential biomarker towards the evaluation of its acute toxic potential. The main effects of THC in the central nervous system are mediated through cannabinoid receptors CB1 densely concentrated at brain regions [78] but it has also been revealed that THC reduced the synthesis of the neurotransmitter acetylcholine in the hippocampus pointing to its negative effects on cognitive processes [79,80]. However, literature reports make it clear that chronic exposure of old animals to low THC doses resulted with an improvement of neurological deficits. Inhibition of acetylcholinesterase (AChE) by THC is considered to be one of the reasons for this improvement [80]. Based on high lipophilicity and a fused tricyclic structure of THC, some authors hypothesized that it could bind to AChE. Eubanks et al. [81] performed computational modelling of the THC-AChE interactions and demonstrated that THC binds in the allosteric peripheral anionic site (PAS) of AChE. Further studies focused on THC effects on cholinesterases reported inconclusive results. In an in vitro study reported by Srivastava et al. [82], a screening of the activity of human red blood cells (RBC) AChE resulted with no inhibitory effect of the *C. sativa* extracts. Ebuehi and Abey [83] reported a reduction of brain AChE activity in Sprague-Dawley rats after seven weeks of exposure to a diet containing 10% and 5% *C. sativa* chow while Abdel-Salam et al. [77] found increased brain AChE activity in Sprague-Dawley rats that were daily subcutaneously given *C. sativa* extract resin rich on THC for six weeks. At the same time, serum BChE activity was markedly inhibited.

In our study, the application of a single THC dose (7 mg/kg) did not affect cholinesterase activities, either in the plasma, or in brain tissue, 24 h after treatment (Table 2).

## 4. Materials and Methods

### 4.1. Chemicals and Reagents

Δ^9^-tetrahydrocannabinol (Dronabinol; CAS-No. 1972-08-3) was obtained from THC Pharm GmbH (Frankfurt, Germany). Other chemicals and reagents were bought from Sigma-Aldrich Laborchemikalien GmbH (Steinheim, Germany).

### 4.2. Breeding and Housing of Animals

Male Wistar HsdBrlHan rats were supplied from the animal facility of the Institute for Medical Research and Occupational Health, Zagreb (Croatia). Animals were maintained under pathogen-free conditions in steady-state micro environmental conditions, 12 h light/dark cycle, room temperature 20–22 °C and humidity 40–60%, with ad libitum access to standard Good Laboratory Practice (GLP) certified food (complete feed for mice and rats 4RF21, Mucedola, Settimo Milanese, Italy) and tap water. Appropriate enrichment was provided in animal cages. The study was approved by the Ethics Committee of the Institute for Medical Research and Occupational Health, Zagreb, Croatia (approval number: 100-21/16-16, 30 June 2016), and was conducted in accordance with the Directive of The European Parliament and of the Council (2010/63/EU) and the Croatian Animal Protection Law (“Official Gazette”, OG 102/2017).

### 4.3. Experimental Design

A total of 30 rats with an initial body weight from 235 to 249 g were randomly assigned to THC-treated and control groups. Each group comprised five animals. 

THC was dissolved in sesame oil (Bio Primo, Ulm, Germany), and administered per os at a daily dose of 7 mg/kg b.w. [84,85,86] repeatedly for one, three, and seven days. 

For each THC group (1-, 3-, and 7-days exposure), an appropriate control group was kept in parallel. Control rats were administered per os the same volume of the vehicle sesame oil once a day. 

Body weights were monitored on a daily basis and the doses of THC were adjusted accordingly. Survival and clinical signs of intoxication were also regularly assessed. Following each THC administration, we monitored activities of treated rats to observe the level of consciousness, any signs of aggression, scratching, tremors, convulsions, staring coats, etc. At the end of each treatment, the body and brain weights of rats were measured and compared with the initial values. 

The experiment ended 24 h after the last treatment. Rats were sacrificed using an anaesthetic cocktail (Narketan, Vetoquinol UK Ltd., Towcester, United Kingdom, 80 mg/kg b.w.; Xylapan, Vetoquinol UK Ltd., Towcester, UK, 12 mg/kg b.w., i.p.). 

The blood samples were collected in heparinized vacutainers by dissection of carotid artery under general anaesthesia and further processed. Heparinised blood was divided into two portions. The first portion of whole blood was immediately used to prepare slides for the comet assay. The second portion was centrifuged (976× *g*, 10 min, at 4 °C) and the plasma removed. Plasma samples were frozen at −20 °C until biochemical analysis. 

Brain tissue samples were dissected, weighed, cleaned from the adhering matters and washed in cold PBS. They were immediately immersed into cold buffer (75 mmol/L NaCl and 24 mmol/L Na_2_EDTA, pH 7.5), and minced into fine pieces with scissors and glass rod. The obtained suspension of cells was used for preparation of slides for comet assay. Brain samples for biochemical analyses were rinsed with cold PBS, frozen in liquid nitrogen, and stored at –20 °C. Prior to the measurements of oxidative stress parameters, the brain samples were homogenized (100 mg tissue/mL in 50 mmol/L potassium phosphate buffer containing 1 mmol/L EDTA, pH 7.4, and centrifuged at 4425 × *g* and 4 °C for 30 min to obtain a supernatant. Prior to the measurements of cholinesterase activities, the brain homogenates were diluted with 50 mmol/L potassium phosphate buffer to 40 mg/mL.

### 4.4. The Alkaline Comet Assay

In this study, the standard procedure of the alkaline comet assay [87], with minor adjustments was followed. Fully frosted microscope slides (Surgipath^®^, Cambridgeshire, UK) were used to prepare agarose microgels. They were first precoated with 0.6% normal melting point (NMP) agarose. Samples of whole blood (V = 4 µL per slide) and brain cells (V = 10 µL per slide) were mixed with 0.5% low melting point (LMP) agarose which was kept at 37 °C to maintain physiological condition. This mixture was pipetted on slides and protected with an upper layer of 0.5% LMP agarose. After solidification, the microgels were dipped in a lysis buffer (2.5 mol/L NaCl (Kemika, Zagreb, Croatia), 100 mmol/L Na_2_EDTA, 10 mmol/L Tris-HCl, 1% sodium lauroyl sarcosinate, pH 10) with 1% Triton X-100 and 10% dimethyl sulfoxide (Kemika). Lysis step lasted overnight at 4 °C. Microgels were then denatured in alkaline denaturation/electrophoresis buffer (300 mmol/L NaOH (Kemika) and 1 mmol/L Na_2_EDTA, pH > 13) for 20 min. Electrophoresis lasted for 20 min, at 4 °C, 25 V, and 300 mA. To neutralise microgels, Tris-HCl buffer (0.4 mol/L; pH 7.5) was used. Before comet scoring, the gels were stained with ethidium bromide (20 µg/mL). Microscopic analysis was done at 200× magnification under an epifluorescence microscope (Olympus BX50, Tokyo, Japan). The extent of DNA damage in single cells was determined using Comet Assay^TM^ software (version IV, Instem-Perceptive Instruments Ltd., Suffolk, Halstead, UK). The same scorer completed measurements on the coded/blinded slides. Scoring was performed in randomly selected fields, by capturing the comets at a constant depth of the microgel. A total of 200 representative comets per rat/tissue were captured on replicate slides in two independent evaluations (altogether 1000 individual comet measurements per each experimental group were accomplished). To quantify the extent of DNA damage, three parameters were selected: tail intensity (i.e., DNA% in tail), tail length (presented in micrometres) and total area (represents the overall surface area of the comet).

### 4.5. Biochemical Markers of Oxidative Stress

#### 4.5.1. Thiobarbituric Reactive Substances (TBARS) Assay

Concentration of thiobarbituric reactive substances (TBARS) was measured using a modified method proposed by Drury et al. [88]. Plasma/brain tissue homogenate samples (V = 50 µL) were mixed with butylated hydroxytoluene (BHT; 5 µL; 0.2%; *w*/*v*) and phosphoric acid (750 µL; 1%; *v*/*v*). After mixing, 250 µL 0.6% (*w*/*w*) thiobarbituric acid (TBA) and 445 µL H_2_O were added and the reaction mixture was incubated in a water bath at 90 °C for 30 min. After cooling, the absorbance was measured at 532 nm on a Shimadzu UV Probe Spectrophotometer (Shimadzu Corporation, Kyoto, Japan). The concentration of TBARS was calculated using standard curves of increasing 1,1,3,3-tetramethoxypropane concentrations, and expressed as µmol/L.

#### 4.5.2. Ferric Reducing Antioxidant Power (FRAP) Assay

To establish the total antioxidant capacity (TAC), a FRAP assay was used. It depends on the reduction of the Fe^3+^-TPTZ complex under acidic conditions [41]. The reagents comprised 300 mmol/L acetate buffer (pH 3.6) with 16 mL acetic acid per mL of buffer solution, 10 mmol/L 2,4,6-tri[2-pyridyl]-s-triazine (TPTZ) in 40 mmol/L HCl and 20 mmol/L FeCl_3_. To prepare a working FRAP reagent, 20 mL acetate buffer, 2.0 mL TPTZ solution, 2.0 mL FeCl_3_ solution and 2.4 mL distilled water were mixed. Then, 30 µL of plasma/brain tissue homogenate sample was added to 1 mL of freshly prepared reagent warmed at 37 °C. The complex between Fe^2+^ and TPTZ produces a blue colour with absorbance at 593 nm. To obtain the calibration curve, water solutions of known FeSO_4_·7H_2_O concentration, in the range of 0.1–1.0 mmol/L were used.

#### 4.5.3. Glutathione (GSH) Assay

The concentration of reduced glutathione (GSH) was determined in plasma and supernatants of the brain using Ellman’s method [89]. An amount of 850 μL of 0.3 mol/L phosphate buffer, pH 7.4, and 50 μL of 10 mmol/L 5,5′-dithiobis(2-nitrobenzoic acid) (DTNB) were mixed with 100 μL of sample. Absorbance was measured spectrophotometrically at 412 nm, and the concentration of GSH was calculated using calibration curve. The results were expressed as μg/mL.

#### 4.5.4. Superoxide Dismutase (SOD) Activity

Total SOD activity in plasma and supernatants of the brain was measured spectrophotometrically as proposed by Flohé and Ötting [90]. The reduction rate of cytochrome c by superoxide radicals was monitored at 550 nm utilizing the xantine-xantine oxidase system as the source for O_2_^−^. SOD competed for superoxide and decreased the reduction rate of cytochrome c. One unit of SOD was defined as the amount of enzyme that inhibits the rate of cytochrome c reduction by 50%. Enzyme activity was expressed as IU/g_protein_.

#### 4.5.5. Catalase (CAT) Activity

Catalase activity was measured in plasma and supernatants of the brain at 240 nm (25 °C, pH 7.0) [91]. Enzyme activity was calculated using the molar extinction coefficient (40.0 mM/cm) and was expressed as IU/g_protein_.

#### 4.5.6. Protein Quantification

Determination of protein concentration was carried out according to the method of Bradford [92] using bovine serum albumin as standard.

### 4.6. Cholinesterase Activity Assay

Blood and brain tissue samples were analysed for total ChE, AChE and BChE activities using the spectrophotometric Ellman method [93]. Enzyme activity was measured in a 0.1 mol/L sodium phosphate buffer, pH 7.4, at 25 °C using ATCh (1.0 mmol/L) and DTNB (0.3 mmol/L). AChE and BChE activities were distinguished using the BChE-selective inhibitor ethopropazine (20 µmol/L). Increase in absorbance was monitored at 412 nm over 4 min. All of the measurements were performed on a Cecil 9000 (Cecil Instruments Limited, Cambridge, UK) Spectrophotometer. Enzyme activity was expressed as IU/g_protein_.

### 4.7. Statistical Analysis

For statistical calculations, the Dell™ Statistica™ 13.2 software (StatSoft, Tulsa, OK, USA) was used. The data collected after measurements in the alkaline comet assay were first processed using descriptive statistics. Prior to data analysis, log10-transformation was used to normalize the data distribution and equalize variances. Further evaluations were performed by Mann–Whitney U test. For statistical evaluations of biochemical markers, Mann–Whitney U test was also used. The level of statistical significance was set at *p* < 0.05.

## 5. Conclusions

The present study is the first to evaluate and document that THC administered to rats at a dose comparable to those find in illicit preparations was able to provoke low-magnitude DNA damage in white blood cells and brain cells of rats detectable using the alkaline comet assay. This is novel information that was not known before. While in our previous paper [20] we showed that THC produced genotoxic effects in rat hepatocytes after seven days of repeated exposure, this study provides evidence that brain and white blood cells responded with significant increases of DNA damage much earlier than hepatocytes. These results indicate different genome susceptibility of the mentioned cell types, as well as time-dependent differences in DNA damage caused by THC in white blood cells, hepatocytes and brain cells of the exposed rats. Taken together, the results of the comet assay reported in the preceding and present study suggest that brain cells are the most vulnerable to THC exposure at a dose comparable to those found in illicit preparations.

Our results also contributed to existing knowledge with evidence that acute exposure to THC-induced oxidative stress in brain cells but did not disturb ChE activities. Results regarding levels of DNA damage estimated by alkaline comet assay and oxidative stress biomarkers recorded after one-day treatment showed a high level of agreement and correlation. This was especially visible in brain tissue, where high levels of DNA damage were accompanied by significantly increased TBARS levels and slightly increased catalase activity. Even though DNA lesions quantified by the alkaline comet assay (DNA strand breaks and alkali labile sites) are subject to repair, increased levels of DNA damage in brain cells call for concern. THC treatment possibly resulted with other types of DNA damage we cannot detect using the applied form of the comet assay. For instance, a part of DNA damage inflicted after treatment could also be oxidative DNA damage. However, without using specific repair enzymes like formamidopyrimidine DNA glycosylase (FPG) or human 8-oxoguanine DNA glycosylase (hOGG1) with the comet assay one cannot accurately estimate the extent of oxidative DNA damage. Repair of this type of DNA damage is also more complex than repair of single strand breaks or alkali labile sites. Taken together, increased levels of DNA damage measured in brain tissue present threats to neurons in terms of both viability and genome stability, since they do not proliferate and inefficient DNA repair might lead to their progressive loss.

We have to stress that the presented results are valid within the frame of the proposed model, and at this preliminary stage of study they were not intended to provide clinically useful information. Considering that we tested a high THC dose, one could assume that the controlled use of prescribed medical cannabis preparations, which contain much lower levels of THC possibly would not result with detrimental effects at DNA level. However, this could be confirmed only after conducting new experiments. As we focused on exposure to a single THC dose, before drawing general conclusions on THC genotoxicity, further research on in vitro and in vivo models applying a broader dose range with repeated exposure is needed. Furthermore, the metabolic differences between the rat and human metabolism also have to be taken into account before making any conclusion relevant for human risk assessment. Forthcoming studies should focus on other modifications of the comet assay that allow more insight into specific types of DNA lesions, especially oxidative DNA damage and DNA repair pathways, as nucleotide excision and base excision repair. Future studies should also clarify probable epigenetic mechanisms behind DNA damage, aneugenic effects, and possible cell-cycle impairments caused by THC. As recent comprehensive review by Reece and Hulse [44] mentioned THC effects on inhibition of tubulin polymerization, there is also growing concern regarding the potential teratogenicity of the compound, which opens another field worthy of investigation as well.

## Figures and Tables

**Table 1:** The levels of thiobarbituric reactive substances (TBARS), glutathione (GSH), ferric reducing antioxidant power (FRAP), catalase (CAT) and superoxide dismutase (SOD) activity in the plasma and brain tissue of male Wistar rats after 1-day treatment with Δ^9^-tetrahydrocannabinol (THC) and in the respective controls.

Sample/Parameter	FRAP (mmol/L)	TBARS (μmol/L)	GSH (μg/mL)	CAT (IU/g_protein_)	SOD (IU/g_protein_)
**Plasma**					
Control	0.170 ± 0.060	4.329 ± 1.481	142.6 ± 65.9	0.018 ± 0.008	0.188 ± 0.055
	0.153	4.553	127.1	0.019	0.204
	0.119–0.264	2.129–6.210	99.5–257.8	0.007–0.027	0.114–0.247
THC	0.124 ± 0.022	3.057 ± 0.826	106.6 ± 18.6	0.011 ± 0.004	0.128 ± 0.030
	0.129	2.927	104.1	0.009	0.131
	0.092–0.148	2.247–4.435	85.9–137.0	0.007–0.016	0.093–0.171
**Brain**					
Control	0.308 ± 0.094	10.597 ± 4.637	28.027 ± 8.302	0.140 ± 0.009	3.533 ± 0.612
	0.271	12.334	28.377	0.138	3.353
	0.229–0.452	5.349–15.670	14.481–36.202	0.131–0.155	2.877–4.249
THC	0.272 ± 0.047	17.256 ± 1.353 *	38.537 ± 2.293 *	0.159 ± 0.0187	1.409 ± 0.330 *
	0.274	17.093	39.663	0.158	1.437
	0.231–0.346	15.899–19.418	34.950–40.448	0.142–0.190	1.066–1.724

Rats received 7 mg/kg b.w. of THC dissolved in sesame oil and administered per os. Control rats received the same volume of sesame oil as the THC group. There were five rats per group, and all measurements were done in triplicate. First row denotes mean ± standard deviation, second row median, and third row range (minimum – maximum value). * *p* < 0.05 vs. control (Mann–Whitney U test).

**Table 2:** Changes in the total cholinesterase activity (ChE), acetylcholinesterase activity (AChE) and butyrylcholinesterase activity (BChE) in plasma and brain of male Wistar rats after 1-day treatment with Δ^9^-tetrahydrocannabinol (THC) and in the respective controls.

Sample	Plasma			Brain		
Parameter	ChE (IU/g_protein_)	AChE (IU/g_protein_)	BChE (IU/g_protein_)	ChE (IU/g_protein_)	AChE (IU/g_protein_)	BChE (IU/g_protein_)
Control	0.107 0.091–0.141	0.073 0.058–0.097	0.033 0.032–0.067	21.8 13.7–24.1	19.7 10.4–20.9	1.4 1.2–4.4
THC	0.104 0.090–0.135	0.073 0.056–0.102	0.041 0.002–0.062	18.1 16.0–21.1	16.5 13.7–19.5	1.6 0.5–2.4

Rats received 7 mg/kg b.w. of THC dissolved in sesame oil and administered per os. Control rats received the same volume of sesame oil as the THC group. There were five rats per group, and all measurements were done in triplicate. The results are shown as median (first row) and ranges (minimum-maximum value; second row).
